# Prevalence, Genetic Structure and Antifungal Susceptibility of the *Cryptococcus neoformans/C. gattii* Species Complex Strains Collected from the Arboreal Niche in Poland

**DOI:** 10.3390/pathogens11010008

**Published:** 2021-12-22

**Authors:** Magdalena Florek, Agnieszka Korzeniowska-Kowal, Anna Wzorek, Katarzyna Włodarczyk, Maja Marynowska, Aleksandra Pogorzelska, Maria Brodala, Sebastian Ploch, Daniel Buczek, Katarzyna Balon, Urszula Nawrot

**Affiliations:** 1 Department of Pathology, The Faculty of Veterinary Medicine, Wrocław University of Environmental and Life Sciences, Norwida 31, 50-375 Wroclaw, Poland; maja.marynowska@upwr.edu.pl (M.M.); aleksandra.pogorzelska@upwr.edu.pl (A.P.); 113077@student.upwr.edu.pl (M.B.); 2 Department of Immunology of Infectious Diseases, Hirszfeld Institute of Immunology and Experimental Therapy, Polish Academy of Sciences, St. Weigla 12, 53-114 Wroclaw, Poland; agnieszka.korzeniowska-kowal@hirszfeld.pl (A.K.-K.); anna.wzorek@hirszfeld.pl (A.W.); 3 Department of Pharmaceutical Microbiology and Parasitology, Wrocław Medical University, Borowska 211a, 50-556 Wroclaw, Poland; katarzyna.wlodarczyk@umed.wroc.pl (K.W.); danielbuczek@onet.pl (D.B.); katarzyna.balon@hirszfeld.pl (K.B.); urszula.nawrot@umw.edu.pl (U.N.); 4 IT Lab, The Faculty of Veterinary Medicine, Wrocław University of Environmental and Life Sciences, Norwida 31, 50-375 Wroclaw, Poland; sebastian.ploch@upwr.edu.pl; 5 Laboratory of Genomics & Bioinformatics, Hirszfeld Institute of Immunology and Experimental Therapy, Polish Academy of Sciences, 53-114 Wroclaw, Poland

**Keywords:** *Cryptococcus neoformans/C. gattii* species complex, environmental, arboreal, RFLP, MALDI-TOF MS, susceptibility

## Abstract

Fungi belonging to the *Cryptococcus neoformans/C. gattii* species complex (CNGSC) are etiological agents of serious and not infrequently fatal infections in both humans and animals. Trees are the main ecological niche and source of potential exposition concerning these pathogens. With regard to epidemiology of cryptococcosis, various surveys were performed worldwide, enabling the establishment of a map of distribution and genetic structure of the arboreal population of the CNGSC. However, there are regions, among them Central and Eastern Europe, in which the data are lacking. The present study shows the results of such an environmental study performed in Wrocław, Poland. The CNGSC strains were detected in 2.2% of the tested trees belonging to four genera. The obtained pathogen population consisted exclusively of *C. neoformans*, represented by both the major molecular type VNI and VNIV. Within the tested group of isolates, resistance to commonly used antimycotics was not found, except for 5-fluorocytosine, in which about 5% of the strains were classified as a non-wild type.

## 1. Introduction

Cryptococcosis is a serious, not infrequently fatal infection of humans and animals caused by fungi belonging to the genus *Cryptococcus*. Though several species of the genus were found responsible for the disease occurrence [1], those belonging to the *Cryptococcus neoformans/Cryptococcus gattii* species complex (CNGSC) were the etiological agents of the majority of cases. The worldwide impact of cryptococcosis in the human population only is assumed to be more than 220,000 infections and up to 180,000 deaths annually [2]. The disease induced by *C. neoformans* is usually observed in persons with impaired immune status, while *C. gattii* is thought to be able to cause infection in immunocompetent individuals; yet, there are reports suggesting genetically mediated differences both in host and pathogen may affect this typical epidemiological pattern [3]. 

The nomenclature concerning the CNGSC has been changing during the previous and ongoing century. These pathogens can be described by their species, major molecular type, or serotype. With respect to the species, presently, the two-species or seven-species schemes relating to the complex are of use, as the discussion concerning classification of the fungi did not reach a consensus among the scientific community [3,4]. According to the consensus [5] based on various molecular methods, the described fungal complex has been divided into eight major molecular types (MMT), and lately, a ninth one has been discovered [6]. The fungi can be also divided into four serotypes, namely A, B, C, or D. In the present paper, the nomenclature with regard to a two-species scheme as well as division into nine MMT were adopted [4,5,6]: VNI, VNII, and VNB (all representing *Cryptococcus neoformans* var. *grubii*, serotype A); VNIV (*C. neoformans* var. *neoformans*, serotype D); and VNIII (the hybrid of these two varieties, serotype AD) as well as VGI, VGII, VGIII, VGIV, and VGV (*C. gattii*, serotype B or C). 

The discussed fungi are in possession of a bipolar mating system consisting of a single mating-type locus *(MAT*) represented by two different mating types (*MAT*a and *MAT*α). Both bisexual and unisexual mating was observed within the CNGSC [7,8].

With respect to the geographical distribution of pathogens belonging to the described complex, *C. neoformans* seems to be global, while *C. gattii* was regarded as associated rather with tropical and subtropical zones [9]. For about the last twenty years, however, the latter one has also been detected in regions with typically temperate climate [10,11]. 

It is assumed that infection with fungi of the CNGSC is a result of inhalation of spores or dehydrated yeast cells and is acquired from environmental sources, such as plant materials, soil, or bird excreta [12,13]. Arboreal sources are considered the main natural niche [13] of the fungi, and according to a growing number of available publications, the presence of CNGSC in certain parts of the world is often associated with specific species of flora [13,14,15,16]. Interestingly, in some surveys comparing clinical and environmental strains within the same area, it was impossible to recognise the environmental sources of those clinical strains [17,18,19,20]. Moreover, when compared on a genetic level, these two groups of strains often show discrepancies [21]. While some of those discrepancies may be regarded as the result of dormant infections imported from other areas of the world, another may indicate the presence of indigenous, as-yet-unrevealed sources of the pathogen [22]. Importantly, it was also observed that genetic variability among the CNGSC strains (e.g., among different MMTs) may influence their virulence and susceptibility to antifungals [23,24]. Taking into consideration the results of the above-mentioned reports, both extensive analysis of the prevalence of the pathogen within an environment as well as analysis of the genetic structure of its local population may deepen our understanding of the epidemiology of cryptococcosis. Nowadays, such investigations may grow in importance, as epidemiological studies regarding SARS-CoV-2 infections show that insufficiency of the respiratory system and immunological impairment observed in COVID-19 patients increase the risk for opportunistic fungal infections. In the paper of Song et al. [25], *Cryptococcus* was indicated among the top potential agents causing fungal co-infections in COVID-19 patients. Despite the seriousness of cryptococcosis, the data concerning the occurrence of the agent and its population structure in Poland were not very well documented to date. In our previous study, the population of non-clinical strains of animal origin isolated in Poland was analysed [26]. With the present research, we would like to extend the knowledge of the Polish community of the CNGSC, this time by analysing isolates obtained from the arboreal specimen.

## 2. Results

**Figure 1:**
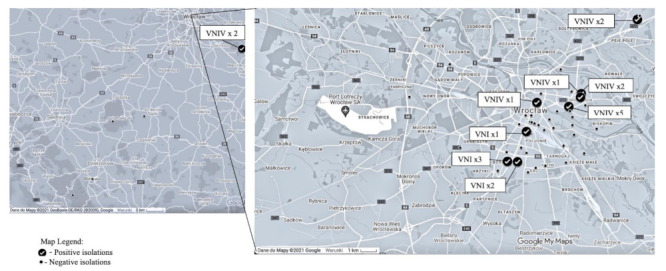
Locations of sample collection points and positive isolation sites (with number of isolates and their MMT).

There were 13 (2.16%) positive outcomes for the presence of the CNGSC strains out of 602 tested samples. With reference to arboreal material, the fungi were detected within specimens collected from 12 trees, among them 9 oaks, as well as one specimen each from birch, pine, and Douglas fir. Statistically significant differences considering the isolation frequency were not detected with regard to tree species (*p* = 0.227). The fungal strains were also cultivated from one of the tested soil samples, which was collected around one of the positive trees. The detailed information concerning the results of isolation of CNGSC are given in Table 1 and Figure 1. Eighteen strains of the CNGSC were isolated from the examined trees. In two cases, multiple strains were detected simultaneously, while other isolates were obtained alone. Since two of the trees were swabbed more than once, a few strains had the same source, yet they were collected on different occasions. One isolate was cultivated from the soil sample.

All the 19 obtained strains were identified as *C. neoformans*. According to the *URA*5-RFLP method, the majority of them (11; 57.9%) represented the MMT VNIV, while the rest (8; 42.1%) belonged to the MMT VNI. One of the MMT VNIV strains was the bearer of a point mutation giving it an atypical *URA*5-RFLP banding profile [27]. Neither strains of the MMT VNII nor AD hybrids were observed within the group of isolates. The obtained serotyping results were in agreement with those obtained in the MMT identification, and all the strains were assigned as representing mating type α.

Applying the MALDI-TOF MS method, all the tested strains achieved scores above 1.7 (regardless of whether the original base or its supplemented version was used), which enabled reliable recognition. With respect to the manufacturers’ base alone, scores of eight isolates made it possible to recognise a possible genus, and another 10 strains were recognised on the level of secure genus and probable species. Only one isolate exceeded the score of 2.3 and was identified as a highly probable species. The mean score secured within the tested group was 2.051, with this value slightly higher among strains belonging to the MMT VNI (2.154) when compared to the MMT VNIV (1.976). Using the best-match strains’ MMTs for comparison, all the tested strains were assigned to MMTs that remained in accordance with results of the other used methods. With the supplemented version of the base, the score values obtained for seven strains (36.8%) increased, resulting in a shift of four of them to the groups with the higher threshold, enabling more secure identification. The mean score obtained for the whole tested group of strains using the updated tool was 2.120, whereas the value concerning the MMT VNI remained almost the same (2.157), and for the MMT VNIV (2.093), it increased enough to exceed the threshold value of the more secure recognition group (2.0–2.3). Considering identification of the MMTs, however, two among the tested strains (10.5%) were misrecognised, as their best-match strains’ MMTs were MMT VNIII instead of the MMT VNIV. The details concerning results of *URA*5-RFLP, sero-, and mating type and MALDI-TOF MS analyses are given in the Table 2.

The result of drug-susceptibility tests of the investigated environmental CNGSC isolates are presented in Table 3.

All the tested isolates were classified as susceptible to amphotericin B (AMB) with a MIC ≤ 1 mg/L [28]. The median value of MICs of AMB was 1 mg/L (range 0.125–1 mg/L) for the whole tested population of isolates as well as for the subgroups representing the MMT VNI (range 0.5–1 mg/L) and the MMT VNIV (range 0.125–1 mg/L). In the case of 5-fluorocytosine (5-FC), the MICs ranged from 1 to 64 mg/L, except of the MMT VNI, for which it was in the range of 8–32 mg/L. The median value of MICs for 5-FC was 16 mg/L, and all the isolates except one (18/19; 94.7%) were classified as belonging to the WT population (MIC ≤ 32 mg/L). 

The MIC values established for triazole derivatives ranged from 0.5 to 32 mg/L for fluconazole (FLU), from 0.03 to 0.25 mg/L for voriconazole (VOR), from 0.015 to 0.25 mg/L for isavuconazole (ISV), from 0.03 to 0.5 mg/L for itraconazole (ITR), and from 0.015 to 0.5 mg/L for posaconazole (POS). The MIC50 for FLU, VOR, ISV, ITR, and POS was 4.0, 0.06, 0.06, 0.125, and 0.125, respectively, whereas MIC90 amounted to 32, 0.25, 0.125, 0.25, and 0.25, respectively. According to adopted ECVs for triazoles, all but one isolate were classified as belonging to the WT population. The only exception was strain FW2 (3168), which showed an ISV MIC of 0.25 mg/L, which is one dilution above the adopted ECV (0.125 mg/L). Regarding statistically significant differences concerning the resistance to specific antimycotics, when compared, the MMT VNI and the MMT VNIV were not detected.

## 3. Discussion

Though pathogenesis of the cryptococcal infections has been extensively examined over the last decades, knowledge considering the environmental origin of the agent remains less well established. In the review presented by Cogliati [29], among an analysis of 69 thousand globally reported strains of the CNGSC, less than 10% represented those of environmental or veterinary origin. The detailed studies over the sources of this environmentally distributed complex are essential in order to define its geographical extent, population structure, and mode and risk of transmission. It was also proven that certain environmental conditions, among them those associated with arboreal ancestry of these fungi, may be responsible for the acquisition of virulence factors allowing them to establish infection within human/animal hosts [30,31,32,33,34]. In Europe, several studies concerning the arboreal source of the CNGSC were performed, the majority of which were related to western and southern regions of the continent as well as to Mediterranean Basin [13,17,35,36,37,38]. Contrastingly, in Central and Eastern Europe, investigations concerning the matter, to the best of our knowledge, are almost absent [39].

In Poland, only a handful of examinations with reference to the environmental prevalence of the pathogenic complex were carried out. *C. neoformans* has been detected in 35.3% of the tested sandpits and 19% of soil samples obtained from children’s recreational areas in Łódź, a city located in the central of Poland [40,41]. The fungus was also recognised in the waters of Charzykowskie Lake and its influents as well as in Szczecin Lagoon, both located in the north of the country [42,43]. In the present study, an analysis of arboreal specimen collected in the southwestern part of Poland was performed. The CNGSC isolates were obtained from 12 out of 592 (2.2%) trees tested as well as from one in ten soil samples collected in the trees’ proximity. The positive trees represented genera *Quercus*, *Betula*, *Pinus,* and *Pseudotsuga*; yet, none of these were associated with a significantly higher frequency of isolation of the fungi. The prevalence of the CNGSC in the arboreal sources in Europe varied substantially. In the ISHAM’s project [13], surveying samples collected in nine European and three non-European countries, the CNGSC strains were recovered from 5% of the trees. Similar results (4.8%) were reported by Montagna et al. [17] with regard to Southern Italy. A slightly higher number was presented by Romeo et al. [36] again in Southern Italy, as the authors reported nine positives among 143 trees tested. In the Netherlands, depending on the study, the fungi were not recovered at all [13], or the isolation frequency amounted to 3.8% (2 of 52 trees) [11]. A ratio far higher, reaching about 16%, has been noticed in Spain (1 of 6 trees) [35] and in Greece [13]. In Portugal, the CNGSC was isolated from 3 out of 28 samples [38]. However, prevalence lower than that observed in our study was noted in Croatia [37] (4 in 472 samples) and Central Italy [44] (1 in 265 trees). Negative results in the context of arboreal specimen were reported in Russia [39] and Germany [13]. The observed discrepancies were probably related to the climate conditions within the tested zones, species composition of the used tree population, or number of the samples tested [13,37,45,46]. It was determined [45,46] that distribution range of certain species belonging to the CNGSCN in the environment is strongly correlated with climatic conditions, such as minimum/mean temperature in the coldest season/quarter, maximum temperature, summer rainfall/precipitation in the driest month, water vapor pressure, solar radiation, distance from the coast, and canopy closure [45,46,47,48]. With respect to European arboreal niche, *C. neoformans* has been isolated from trees belonging to the following genera: *Eucaliptus*, *Olea*, *Pinus*, *Creatonia*, *Pyrus*, *Prunus*, *Platanus*, *Aesculus*, *Carpinus*, *Juglans*, *Juniperus*, *Gleditsia*, and *Quercus*, representing both Mediterranean and temperate climate regions [17,35,36,45]. Regarding *C. gattii*, the fungus has been obtained from trees of the following genera: *Eucaliptus*, *Olea*, *Creatonia*, *Pinus*, *Pseudotuga* [14,17,35,36,45]. It was suggested that *C. gattii* prefers trees with waxier cuticles [49]. In the matter of prevalence, it was observed [13] that the percentage of isolation of *C. gattii* was statistically higher pertaining to the genus *Creatonia*. *C. neoformans* var. *grubii* has been isolated statistically more often from the *Creatonia* and *Olea*, while *C. neoformans* var. *neoformans* as well as the hybrid of these two have been obtained mainly from the genus *Platanus*. None of the 36 tested trees of the genus *Platanus* swabbed in our study were positive with regard to presence of the CNGSC.

In the present study, 57.9% of the isolated fungi represented the MMT VNIV, while the rest (42.1%) belonged to the MMT VNI. Other molecular types, among them those assigned to the species *C. gattii*, were not detected. Results of surveys concerning arboreal sources of the fungi performed in Europe showed predominance of the MMT VNI. The type constituted from 64.45% (330 of 512 total strains isolated) to 100% of the populations analysed in various studies [11,13,17,36,38,44]. To the contrary, the MMT VNIV was often absent [11,17,36,38,44] or was detected in a percentage ranging from 20.9 (107 of 512) to 25 [13,37]. The high frequency of isolation of the MMT VNIV in the present study remains, however, in accordance with results of work reported by Cogliati et al. [45], predicting a fundamental niche for this MMT as positioned in the sub-continental region of Europe and not along the coast, which corresponds with the location of Wrocław. Of interest may be the observation that, while comparing populations of non-clinical/environmental strains isolated from animal and arboreal specimen within the same area, those of animal origin were reported to show a higher frequency of isolation of the MMT VNIV. In northern Portugal, [38] from arboreal specimens, the MMT VNI was obtained exclusively, whereas 15 in 23 isolates obtained from the samples originated from pigeons represented the MMT VNIV (while the rest was equally distributed between the MMTs VNI and VNIII). Similarly, in our previous study [26] among the non-clinical strains of animal origin, the MMTs VNIV, VNI, and VNIII were represented by 74.36%, 15.38%, and 10.26% of isolates, respectively. It was observed that in Europe, the MMT VNIII was detected more frequently compared to other continents [29]. While among clinical strains, AD hybrids could comprise about 30% [50], and it was not uncommon to isolate the type from specimen of animal origin [26,38], to the best of our knowledge, the only European arboreal isolation of the MMT VNIII was reported in Greece [13]. Another rare type, the MMT VNII, has not been isolated from trees in Europe to date. *Cryptococcus gattii* is the species that has been reported as occurring in different genera of trees of southern Europe [13,17,36]. It was also isolated in the temperate climate zone of northern Europe; the pathogen inhabited one Douglas fir tree found in the Netherlands [11]. With respect to the MMTs, in arboreal specimens collected in Europe, *C. gattii* was represented by the MMT VGI and the MMT VGIV [11,13,17,36].

With respect to mating type, within the population of the CNGSC, the *MAT*α locus is regarded as the most prevalent both among clinical and environmental strains [29], whereas *MAT*a is rather rare, and it is more commonly observed within the MMT VNIV strains [51]. All the strains isolated in the present study, regardless of their serotype, represented mating type α. Similar to our work, populations of the CNGSC isolated in Europe from tree material consisting purely of mating type α were reported [36,37]. On the other hand, in the study performed by Montagna et al. [17], the locus *MAT*a was detected in one of 40 strains of the serotype A, whereas in the survey presented by ISHAM [13], the locus was detected in 7 of 330 isolates representing serotype A, 29 of 107 belonging of the serotype D, and in 3 out of 35 AD hybrids (αADa). It is worth mentioning that within the population of the strains isolated from animals in Poland, locus *MAT*a was found in aD or aADa isolates [26]. Among the arboreal strains of *C. gattii* isolated in Europe, researchers observed sero- and mating types αB, aB, and αC [11,13,17,36].

The population of the yeasts investigated in the present study showed a low rate of resistance to the tested antimycotics. According to the criteria adopted, all the CNGSC isolates were classified as susceptible to AMB or belonging to WT-population in regard to the susceptibility to FLU, ITR, POS, and VOR. Similar results were presented in our previous study [26] in which, among the CNGSC isolates obtained from asymptomatic animals (mostly pigeons), triazole resistance was not detected. Comparison of the results obtained by other authors with respect to the CNGSC susceptibility to triazole is difficult. In those studies, in which we could find data concerning strains of arboreal origin, those were rarely presented alone [15,37,52], and the overall data presenting the matter were sparse. Often, mixed results for both plant- and pigeon-derived specimens [53] or soil contaminated with human/animal material were presented [54]. Moreover, applied interpretation criteria or different testing methods made it impossible to compare the results even by means of the MIC values. As an example, authors analyzing the CNGSC strains in Croatia using the ATB fungus test found all the population susceptible to FLU, ITR, and VOR, while in Brazil (AFST-EUCAST), 78.9% of the obtained isolated were classified as non-WT with regard to FLU [15,37]. Interestingly, in both the present and our previous study, high MIC values were obtained for 5-FC. According to the applied ECV (32 mg/L; Cordoba et al. [55]), in the present study, 1 out of 19 isolates (5%) was classified as resistant (or rather as non-WT) to this drug (Table 3). If applied to the epidemiological cut-off proposed by Espinel-Ingroff [56], however, four other strains with the MIC amounting to 32 mg/L (three MMT VNI and one MMT VNIV) could also be listed as non-WT. The number of non-WT strains detected according to the former ECV in the present study was lower compared to the results reported in our previous paper [26], in which 10% of the strains showed the MIC values for 5-FC equal to 64 mg/L. The reason for the observed discrepancy between arboreal and animal isolates was probably the size of both studied groups (19 vs. 39), and statistically significant differences were not confirmed. It should be emphasized that those resistant to 5-FC strains evaluated in our studies (four of animal and one of arboreal origin) were collected in different locations and over subsequent four years; thus, they rather were not related epidemiologically. Of importance may also be the fact that all of these isolates represented the same MMT (VNIV). With regard to 5-FC, the reports of other authors showed that the resistance among clinical and environmental *Cryptococcus* isolates was rather low (1–2.5%) [55,56,57,58] or undetectable [59]. Interestingly, in their work, Chowdhary et al. [59], while presenting low MIC values for 5-FC regarding both clinical and environmental strains, estimated that environmental isolates (in this case arboreal) *C. neoformans* var. *grubii* presented significantly reduced susceptibility compared to clinical strains of the same variety. Although the primary resistant strains identified in our studies were rare (5–10%), the phenomenon is of particular concern, as 5-FC is a drug recommended for treatment of cryptococcal meningitis. Due to the severity of this type of infection and the possibility of a fatal outcome, the therapy is usually introduced in an empirical or preemptive manner before obtaining the final results of microbiological examination and susceptibility tests. Therefore, knowledge with regard to the prevalence of resistant strains within the local environment may enhance the selection of the most potent therapy. 

The significant impact of factors, which may be present in the natural habitat of the CNGSC, including nutrient limitation (among them nitrogen limitation), temperature, ultraviolet radiation, enzymatic degradation on the susceptibility of the CNGSC to FLU, and AMB, has been described [34,52,60,61,62,63]. The above-listed factors are probably able to activate adaptive processes, favoring the survival of the microorganism in the presence of the drugs; however, their role in the development of persistent, mutation-dependent resistance cannot be excluded. Additionally, it has been documented that both antifungal and non-antifungal agrochemicals may exert a similar effect on environmental strains of the CNGSC [64,65]. To the best of our knowledge, however, neither use of agrochemicals nor recognized mechanisms of the resistance [66,67,68] can explain environmental sources of the CNGSC resistance to 5-FC. Nevertheless, detection of the primary resistant strains may suggest existence of as yet not recognized environmental factors supporting the development of their resistance.

It is possible that, for the sake of the small isolate number obtained in the present study, it was impossible to detect statistically significant differences in the MIC distributions of any of the tested drugs with regard to MMTs of the tested strains. On the contrary, our previous work results proved that the average observed MIC value of amphotericin B was significantly lower and of fluconazole was significantly higher for the MMT VNIV compared to the MMT VNI among strains isolated from animals in Poland [26]. Similar significant differences in the drug-susceptibility among particular MMTs of the CNGSC were described by other authors [59,69].

Due to the effort of several groups of researchers documenting the presence of arboreal CNGSC strains within various European regions, it was possible to predict the niche of the particular species within the complex and assess potential areas of exposure [45,46]. According to estimations presented by Alaniz et al. [46], the total area of distribution of these fungi in Europe covers 2.7 million km^2^. Since the ranges of particular species within the complex differ slightly, the authors suggest that the number of people potentially exposed to infection on this continent may reach about 360, 266 and 137 million with reference to *C. neoformans* var. *grubii*, *C. neoformans* var. *neoformans,* and *C. gattii*, respectively. Yet, in order to calculate the risk, environmental surveys must firstly be performed. Unfortunately, the data covering Central, Eastern, and Northern parts of Europe, to the best of our knowledge, remain unavailable. With respect to regions characterised by continental climate, to date, the only available studies were performed in Russia (Saint Petersburg), Germany, and the continental part of Croatia, where analysis of arboreal specimens gave negative isolation results [13,37,39]. Therefore, we believe results of the present study may contribute to knowledge concerning ecology of the CNGSC in Europe.

There is still need for ongoing surveillance concerning environmental presence of the CNGSC and not only with regard to those regions that were not examined to date [70]. With phenomena such as global warming or increasing reduction in biodiversity, changes in the ecology of these fungi [71,72] may be expected, subsequently influencing people at risk.

## 4. Materials and Methods

### 4.1. Study Design and Sample Processing

The arboreal specimen as well as the soil samples were collected between June 2014 and April 2019 in parks situated on the territory of Wrocław (51°6′36″ N, 17°1′20″ E) as well as in forests located within the radius of 60 km from the city (Kotowice, Sobótka). The territory is classified Dfb (warm-summer humid continental climate), according to Köppen–Geiger classification. The material was obtained by the swabbing of tree hollows or by collecting samples of woody detritus/soil. Usually, one sample was collected from one tree (with the exception of one of the oaks, which was swabbed multiple times, and one pine swabbed twice) or from the soil of the tree surroundings. A total of 592 samples of arboreal specimen and 10 soil samples were obtained. With respect to the arboreal sources, most of the tested samples (309; 52.2%) were collected from oaks, while other genera of the trees were represented by 1 to 66 specimens. The detailed information concerning sources of specimen used in the present study is given in Table 1. 

All the samples were vortexed with 20 mL of a sterile saline solution for 5 min and then left in order to let the suspension settle. The obtained supernatants were diluted 1:10. Two sets of plates containing Niger seed agar (NSA) were inoculated with (100 µL) the supernatant or its dilution. Then, the plates were incubated at 30 °C for up to 14 days although positive samples could usually be detected at 48–96 h. All colonies showing the brown colour effect obtained from each sample were sub-cultured as single-colony isolates on NSA in order to purify cultures and then assessed using India Ink staining. Strains positive in morphological evaluation and able to produce melanin on NSA were classified as the CNGSC. A selection of colonies cultured from the same sample for further tests was performed by means of analysis of the colony morphology and melanisation pattern. 

Statistical analyses of isolation frequency with regard to certain tree species were performed using Fisher’s exact test and PQStat v.1.8.2.208 (PQStat Software 2021) software. In each analysis, a significance level of 5% was adopted.

### 4.2. Molecular Examination/Genotyping

For the DNA isolation, the obtained CNGSC strains were cultured on Sabouraud dextrose agar (SDA) for 48 h at 30 °C. Extraction of the DNA was obtained using a MasterPure Yeast DNA Purification Kit (Epicentre Biotechnologies, Madison, WI, USA), in accordance with the manufacturer’s instructions. All PCRs presented in this study were carried out in an MJ Mini Personal Thermal Cycler (BIO-RAD, Hercules, CA, USA) utilising 25-µL reaction volume (1 µL of the extracted DNA, 12.5 µL of master mix (PCR Mix, A&A Biotechnology, Gdynia, Poland), 20 pM of each primer, and 11.1 µL of water). For each reaction, both positive and negative (sterile water) controls were used.

Recognition of species and/or variety was performed by sequencing of the SOD1 gene [73]. Amplification of the gene in *C. gattii* and *C. neoformans* var. *grubii* (MMT VNI) was executed by applying two separate sets of primers presented in the MLST consensus scheme [5]. For *C. neoformans* var. *neoformans* (MMT VNIV), an alternative reverse primer for the SOD1 gene described by Sanchini et al. [74] was employed. 

Sero- and mating types of the tested strains were established using the PCR-based method (amplification of the serotype-specific and mating-type-specific *STE*20 gene) described by Li et al. [75]. The following strains were used as positive controls: CBS 10084 (Aα), CBS 132 (αADa), IUM 96-2828 (Aa), and CBS 10079 (Dα).

Restriction fragment-length polymorphism analysis of the orotidine monophosphate pyrophosphorylase gene (*URA*5-RFLP) was conducted according to Meyer et al. [76]. The obtained PCR products were double digested using *Cfr*13I (Sau96I) and *Hha*I enzymes (Thermo Fisher Scientific, Waltham, MA, USA) for 16 h and then separated in 3% agarose gel (100 V for 3 h). RFLP patterns of the tested strains were analysed visually by comparison with banding characteristic for standard strains representing major molecular types (CBS 8710-VNI, CBS 10084-VNII, CBS 132-VNIII, and CBS 10079-VNIV). 

### 4.3. Identification with the Use of MALDI-TOF MS Method

Matrix Assisted Laser Desorption/Ionization Time-of-Flight Mass Spectrometry (MALDI-TOF MS) analysis was performed on ultrafleXtremer spectrometer (Bruker Daltonics GmbH, Germany), as described in our previous study [26]. Biotyper 3.1 software (Bruker Daltonics GmbH, Germany) and an in-house supplemented [26] manufacturer’s database (8469 entries) was used for the isolates’ identification. Manufacturer given score values were used: <1.7 (identification not reliable), 1.7–2.0 (probable genus identification), 2.0–2.3 (secure genus identification and probable species identification), and >2.3 (highly probable species identification). The highest scores among the series of repetitions were given as the result. In order to define the major molecular type of the examined strains, the best match strains’ MMTs were analysed.

### 4.4. Susceptibility to Antifungal Drugs

The susceptibility of CNGSC isolates to amphotericin B (AMB), 5-fluorocytosine (5-FC), fluconazole (FLU), isavuconazole (ISV), itraconazole (ITR), posaconazole (POS), and voriconazole (VOR) was tested using the microdilution method, according to the European Committee on Antimicrobial Susceptibility Testing (EUCAST) Definitive Document E.DEF 7.3.1. [77]. All applied reagents were purchased from Sigma-Aldrich Life Science. The minimal inhibitory concentration (MIC) definition was the lowest drug concentration resulting in 90% (AMB) or 50% (5-FC and triazole derivatives) reduction of the OD530 when compared to the drug-free control. 

The clinical breakpoints as well as epidemiological cut-off values (ECVs) applied in the present study were consistent with our previous work [26] and amounted to for amphotericin B (1 mg/L), POS (0.5 mg/L), and VOR (0.5 mg/L), 32 mg/L for 5-FC and FLU, 0.5 mg/L for ITR, and 0.125 mg/L for ISV [28,55,78,79]. According to the clinical breakpoints, strains were identified as susceptible or resistant, and using ECV’s values, it was possible to categorise the isolates into wild-type (WT; population of isolates in a species-drug combination with no detectable acquired resistance mechanisms [78]) or non-wild-type (non-WT; strains that may hold mutation).

In order to compare the distribution of MICs between particular MMTs, the Mann–Whitney U test was applied using PAST for Mac OS X v.4.0 (Øyvind Hammer 1999–2021) software. In each analysis, a significance level of 5% was adopted.

## Figures and Tables

**Table 1:** The results of isolation of CNGSC strains from environmental sources.

Source of the Sample	No. of Trees	No. of Positive Trees	Isolation Percentage (%)	No. of Isolates	Strains
Oak (*Quercus* L.)	309	9	2.91	14	1^o^*, 4’, 9a, D4, D2a^, D2b^, K1a^, K1b^, Fw1, Fw4*, Fw5, Fw6, Fw7*, Fw8*
Black locust (*Robinia pseudoacacia* L.)	23	0	0	0	-
Lime tree (*Tilia* L.)	11	0	0	0	-
Plane tree (*Platanus* L.)	36	0	0	0	-
Horse-chestnut (*Aesculus hippocastanum* L.)	12	0	0	0	-
Hornbeam (*Carpinus* L.)	10	0	0	0	-
Birch (*Betula* L.)	10	1	10	1	Fw3
Pine (*Pinus* L.)	13	1	7.69	2	Fw2*, Fw9*
Fir (*Abies* Mill.)	6	0	0	0	-
Willow (*Salix* L.)	4	0	0	0	-
European beech (*Fagus sylvatica* L.)	5	0	0	0	-
Common yew (*Taxus baccata* L.)	2	0	0	0	-
Maple (*Acer* L.)	30	0	0	0	-
Swedish whitebeam (*Sorbus intermedia* L.)	2	0	0	0	-
Sycamore maple (*Acer pseudoplatanus* L.)	2	0	0	0	-
Sea buckthorn (*Hippophaë rhamnoides* L.)	1	0	0	0	-
Thuja (*Thuja* L.)	10	0	0	0	-
Cottonwood (*Populus* L.)	10	0	0	0	-
Larch (*Larix* Mill.)	4	0	0	0	-
Spruce (*Picea* A. Dietr.)	8	0	0	0	-
Alder (*Alnus* Mill.)	9	0	0	0	-
Ash tree (*Fraxinus* L.)	9	0	0	0	-
Douglas fir (*Pseudotsuga menziesii)*	66	1	1.51	1	9x
Trees in total:	592	12	2.20	18	
Soil:	10	1	10	1	Fw10
In total:	602	13	2.16	19	-

* strains obtained from the same tree on several occasions; ^ strains obtained from the same trees simultaneously.

**Table 2:** The results of examination of the MMT (using *URA*5-RFLP and MALDI-TOF MS techniques), sero- and mating- types of the CNGSC strains.

The Strain Name	PCM Number	Sero- and Mating Type	MMT According to *URA*5-RFLP	MALDI-TOF MS Original Base		MALDI-TOF MS Extended Base	
				Score	MMT Identification	Score	MMT Identification
1^o^	3145	Dα	VNIV	1.865	VNIV	2.553	VNIV
9a	3146	Dα	VNIV	1.968	VNIV	2.080	VNIV
9x	3147	Dα	VNIV	1.881	VNIV	1.953	VNIV
4’	3150	Dα	VNIV	1.946	VNIV	1.946	VNIV
D2a	3148	Aα	VNI	2.234	VNI	2.260	VNI
D2b	3149	Aα	VNI	2.221	VNI	2.221	VNI
D4	3151	Aα	VNI	2.090	VNI	2.090	VNI
FW1	3153	Aα	VNI	2.112	VNI	2.112	VNI
FW2	3155	Aα	VNI	2.252	VNI	2.252	VNI
FW3	3158	Dα	VNIV	2.212	VNIV	2.212	VNIV
FW4	3160	Dα	VNIV	1.762	VNIV	1.762	VNIV
FW5	3161	Aα	VNI	2.045	VNI	2.045	VNI
FW6	3162	Dα	VNIV	1.963	VNIV	2.032	VNIV
FW7	3152	Dα	VNIV	2.108	VNIV	2.127	VNIII
FW8	3157	Dα	VNIV	2.002	VNIV	2.041	VNIII
FW9	3159	Aα	VNI	1.853	VNI	1.853	VNI
FW10	3154	Aα	VNI	2.426	VNI	2.426	VNI
K1a	3156	Dα	VNIV	1.965	VNIV	1.965	VNIV
K1b	3000	Dα	VNIV*	2.070	VNIV	2.070	VNIV

* strain presenting atypical banding pattern.

**Table 3:** The susceptibility of the investigated CNGSC isolates to antifungal agents in relation to major molecular type (MMT).

The Strain Name	MIC (mg/L)						
	AMB	5-FC	FLU	VOR	ISV	ITR	POS
Major Molecular Type VNI							
D2a	1	16	0.5	0.06	0.03	0.03	0.06
D2b	1	16	1	0.03	0.06	0.03	0.125
D4	1	16	1	0.03	0.06	0.03	0.125
FW1	0.5	32	32	0.25	0.125	0.25	0.25
FW 10	1	32	32	0.25	0.125	0.25	0.5
FW2	1	8	16	0.25	0.25	0.5	0.25
Fw9	1	32	32	0.125	0.015	0. 25	0.015
FW5	0.5	16	16	0.125	0.125	0.25	0.25
Median (range) of VNI	1 (0.5–1)	16 (8–32)	16 (0.5–32)	0.125 (0.03–0.25)	0.0925 (0.015–0.25)	0.25 (0.03–0.5)	0.187 (0.015–0.5)
Major Molecular Type VNIV							
1^o^	1	16	0.5	0.125	0.125	0.03	0.06
9a	0.5	16	0.5	0.06	0.06	0.03	0.06
9x	1	32	0.5	0.06	0.06	0.03	0.06
4	1	16	0.5	0.03	0.06	0.03	0.25
Fw7	0.5	16	16	0.25	0.125	0.25	0.125
K1a	1	8	8	0.125	0.015	0.125	0.015
Fw8	1	16	4	0.125	0.015	0.03	0.015
FW3	0.5	8	4	0.06	0.015	0.03	0.015
FW4	1	8	8	0.06	0.015	0.125	0.015
FW6	0.125	1	4	0.06	0.06	0.5	0.25
K1b	1	**64**	1	0.03	0.03	0.125	0.125
Median (range) of VNIV	1 (0.125–1)	16 (1–64)	4 (0.5–16)	0.06 (0.03–0.25)	0.06 (0.015–0.125)	0.03 (0.03–0.5)	0.06 (0.015–0.25)
Median (range) of VNI and VNIV	1 (0.125–1)	16 (1–64)	4 (0.5–32)	0.06 (0.03–0.25)	0.06 (0.015–0.25)	0.0775 (0.03–0.5)	0.0925 (0.015–0.5)
MIC50 (VNI and VNIV)	1	16	4	0.06	0.06	0.125	0.125
MIC90 (VNI and VNIV)	1	32	32	0.25	0.125	0.25	0.25

Abbreviations: AMB, amphotericin B; 5-FC, 5-fluorocytosine; FLU, fluconazole; ISV, isavuconazole; ITR, itraconazole; POS, posaconazole; VOR, voriconazole.

## Data Availability

All data generated or analysed during this study are included in this published article. The strains used in the present study were deposited into Polish Collection of Microorganisms (PCM) at Hirszfeld Institute of Immunology and Experimental Therapy PAS. The deposit numbers are given in Table 2.
